# Exploring the key clinical and CT characteristics of granulomas mimicking peripheral lung cancers: a case-control study

**DOI:** 10.1186/s13244-025-02043-0

**Published:** 2025-07-19

**Authors:** Hong-bo Xu, Can Ding, Min Zhao, Fa-jin Lv, Zhi-gang Chu

**Affiliations:** Department of Radiology, The First Affiliated Hospital of Chongqing Medical University, Chongqing, China

**Keywords:** Granulomas, Lung neoplasms, Tomography (X-ray computed)

## Abstract

**Objectives:**

Some granulomas exhibit CT manifestations similar to those of peripheral lung cancers (PLCs), often resulting in misdiagnosis. This study aimed to identify the key clinical and CT indicators for differentiating them.

**Materials and methods:**

From October 2019 to July 2024, 204 atypical granulomas (no calcification, satellite lesions, and/or halo sign) and 204 size-matched PLCs manifested as solid nodules (SNs) were retrospectively enrolled. Patients’ clinical, as well as non-enhanced and contrast-enhanced CT data, were evaluated and compared. The enhancement patterns of lesions included no significant enhancement (▵CT value < 15 HU), rim enhancement, enhancement with well-defined necrosis, heterogeneous enhancement, and homogeneous enhancement. The latter two patterns were further divided into mild (15–29 HU), moderate (30–59 HU), and severe (≥ 60 HU) enhancement.

**Results:**

Multivariate analysis revealed that younger age (≤ 63 years) (odds ratio [OR], 5.237; 95% confidence interval [CI], 2.609–10.509; *p* < 0.001), history of diabetes (OR, 9.097; 95% CI: 3.056–27.077; *p* < 0.001), irregular shape (OR, 3.603; 95% CI: 1.594–8.142; *p* = 0.002), lower non-enhanced CT value (≤ 21 HU) (OR, 7.576; 95% CI: 3.720–15.431; *p* < 0.001), and non-moderate enhancement patterns (OR, 50.065; 95% CI: 20.293–123.517; *p* < 0.001) were independent predictors of granulomas. The sensitivity, specificity, and area under the curve of this model were 88.7%, 83.8%, and 0.941 (95% CI: 0.919–0.962) (*p* < 0.001), respectively.

**Conclusions:**

In younger (≤ 63 years) patients with diabetes, an irregular SN displaying lower density (≤ 21 HU) in non-enhanced CT and a non-moderate enhancement pattern should first be considered as a granuloma.

**Clinical relevance statement:**

Distinguishing atypical granulomas from PLCs can be effectively achieved by evaluating the patient’s age, underlying diseases, and the lesion’s shape, non-enhanced CT value, and enhancement pattern. This integrated clinical-CT diagnostic approach could provide crucial insights for guiding subsequent clinical management.

**Key Points:**

- Atypical granulomas and PLCs exhibit high morphological similarity.
- Enhancement patterns of lesions are crucial for differentiating atypical granulomas and PLCs.
- Atypical granulomas typically display irregular shape, lower non-enhanced CT value, and non-moderate enhancement pattern.
- Younger age and a history of diabetes are key clinical indicators of granulomas.

**Graphical Abstract:**

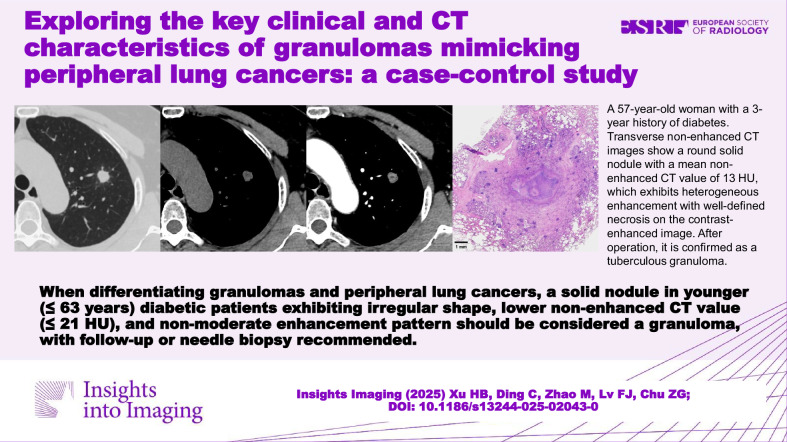

## Introduction

The wide application of computerized tomography (CT) in clinical practice has resulted in a significant increase in the detection of pulmonary nodules [1, 2]. Detected nodules are mainly classified as solid nodules (SNs) and sub-solid nodules (SSNs) based on their density difference. Currently, there have been extensive studies on the radiological identification of SSNs, while the differentiation of SNs remains a significant challenge due to their diverse etiology and complex CT characteristics [3, 4]. Most SNs are benign, and do not require immediate surgical intervention [1]. In contrast, malignant ones are often associated with higher malignant potential and poorer prognosis than SSNs [5, 6]. Thus, accurate diagnosis of SNs is helpful for selecting appropriate treatment options for patients.

Peripheral lung cancers (PLCs) represent the most common malignant SNs, whereas granulomas are a relatively common type of benign SN [1]. A previous study indicated that the CT features between necrotizing granulomas and lung cancers are indistinguishable, and tissue diagnosis is necessary [7]. Furthermore, both granulomas and PLCs were frequently associated with hilar or mediastinal lymphadenopathy [8, 9]. Thus, granulomas are often misdiagnosed as PLCs due to their similarities. Although calcification and satellite lesions are typical indicators in tuberculous granulomas, and halo signs are relatively specific in fungal granulomas [10, 11], there are still a considerable number of granulomas lacking these typical CT features.

Given that atypical granulomas may be more difficult to distinguish from PLCs, it is essential to further differentiate them to guide clinical treatment effectively. However, specific studies focusing on this aspect have not yet been conducted. This study aimed to explore the clinical and CT indicators that can help differentiate between these two entities.

## Materials and methods

### Patients

**Fig. 1:**
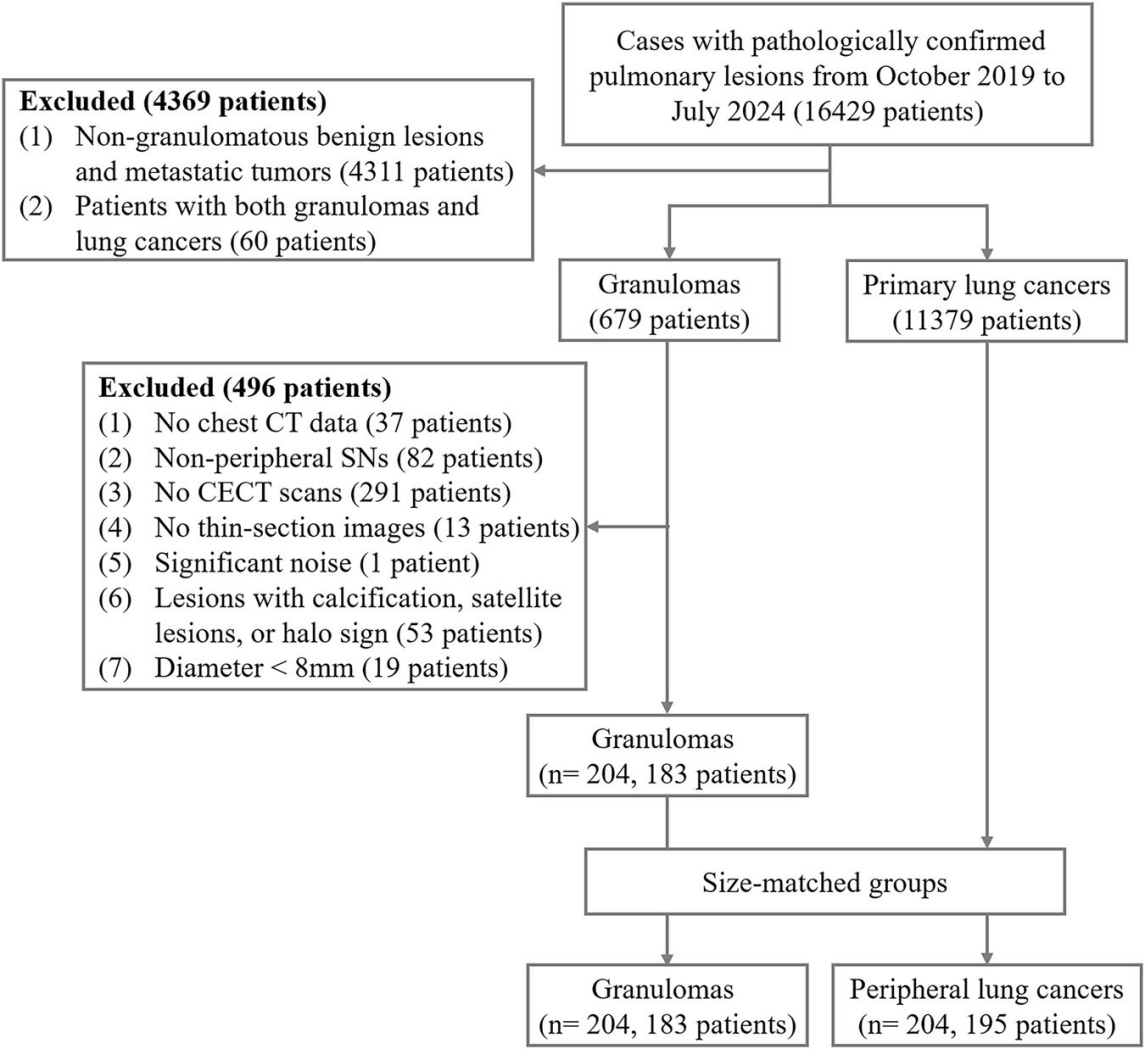
Flow chart of the study patients. CT, computed tomography; SNs, solid nodules; CECT, contrast-enhanced computed tomography

At our institution, incidentally detected indeterminate SNs (≥ 8 mm) are managed according to a standardized diagnostic protocol. Initial management involves a 3-month CT follow-up. Nodules showing growth during follow-up undergo further evaluation with CT-guided core needle biopsy or surgical resection, while stable nodules are assessed by contrast-enhanced CT (CECT) or biopsy. Histologically confirmed malignancies typically proceed to surgical intervention, with PET-CT employed for preoperative staging to assess the extent of disease and guide treatment planning. Biopsy-negative cases with benign CT findings enter extended imaging surveillance. Lesions with discordant results (malignant CT features despite negative biopsy) are managed through multidisciplinary discussion, incorporating patient preferences and clinical risk profiles. Based on this protocol, we retrospectively identified patients who underwent surgical resection of pulmonary lesions from October 2019 to July 2024 through the Pathology Information System (PIS). The enrolled patients required the following conditions to be satisfied: (1) the lesions were confirmed as granulomas or lung cancers by pathological examination, (2) patients had chest CECT scans within two weeks prior to resection, and (3) the lesions were peripheral SNs (diameter ≤ 30 mm) on CT images. The excluded patients required the following conditions to be satisfied: (1) patients with both granulomas and lung cancers, (2) absence of thin-section CT images (≤ 1.5 mm), (3) presence of significant noise on CT images, (4) the nodules had calcification, satellite lesions, or halo sign, and (5) the diameter of the nodules was < 8 mm. The study initially included a final cohort of 183 patients with 204 atypical granulomas manifested as SNs. Considering the potential correlation between the diameter of SNs and their CT features, a control group of PLCs was randomly selected to match the granulomas by size. This was achieved by individually pairing nodules of similar dimensions and applying the same inclusion and exclusion criteria at a 1:1 ratio, resulting in a total of 195 patients with 204 PLCs (Fig. 1).

### CT examinations

The chest CT scans were performed using SOMATOM Perspective (Siemens Healthineers), Discovery CT750 HD (GE Healthcare), SOMATOM Definition Flash (Siemens Healthineers, SOMATOM Force (Siemens Healthineers), and Aquilion ONE pureViSION (Canon Medical System). All patients were placed in a supine position with raised upper limbs and were asked to hold their breath after deep inspiration for better exposure. The scan range was from the thoracic entrance to the costophrenic angle. The following were the scan parameters: tube voltage, 110–130 kVp; tube current time, 50–140 mAs (using automatic current modulation technology); scanning slice thickness, 5 mm; rotation time, 0.5 s; pitch, 1–1.1; collimation, 0.6 mm or 0.625 mm; reconstruction slice thickness and interval, 0.625 or 1 mm; and matrix, 512 × 512. Images of 92.2% of the lesions were acquired using the standardized 120 kV protocol. No significant difference was observed in kV distribution between granulomas and PLCs (110 kV: 13 vs 9, *p* = 0.693; 130 kV: 4 vs 6, *p* = 0.751). All patients underwent CT scans before and after the administration of a nonionic iodinated contrast agent (iopamidol, 3.0 mL/s; 320 mg of iodine per milliliter; 1.2–1.5 mL/kg of body weight; Shanghai Bracco Sine Pharmaceutical Co., Ltd., China). Non-enhanced CT images were acquired before contrast agent administration, while arterial phase (AP) and venous phase (VP) images were obtained at 30 s and 60 s after the injection of contrast agent, respectively. Images were obtained with mediastinal window (width, 400 Hounsfield Unit (HU); level, 30 HU) and lung window (width, 1500 HU; level, −600 HU) settings.

### Clinical and CT data analysis

The patients’ clinical data and laboratory parameters were systematically recorded based on the electronic health record (EHR). The clinical data included the patients’ age, gender, history of hypertension, history of diabetes, history of chronic obstructive pulmonary disease (COPD), history of malignant tumors, smoking history, drinking history, and clinical symptoms. Laboratory parameters included the white blood cell count, neutrophil count, lymphocyte count, monocyte count, carbohydrate antigen 19-9 (CA 19-9), pro-gastrin-releasing peptide (pro-GRP), cytokeratin 19, and carcinoembryonic antigen (CEA). To determine whether they were elevated or not based on the reference ranges of these parameters.

**Fig. 2:**
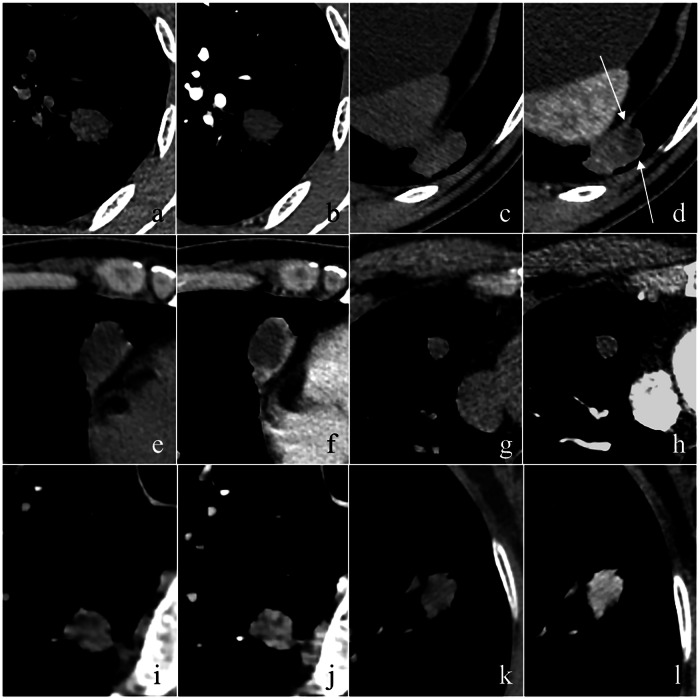
Enhancement patterns of nodules on CECT images with mediastinal window. A 52-year-old man with a tuberculous granuloma. A transverse non-enhanced CT image shows a round SN (12 HU) located in the left upper lobe (**a**), which has no significant enhancement (▵CT value = 0 HU) on the CECT image (**b**). A 40-year-old man with necrotizing granuloma. A transverse non-enhanced CT image shows an oval SN (38 HU) located in the left lower lobe (**c**), which has heterogeneous enhancement with well-defined necrosis (arrows) on the CECT image (**d**). A 50-year-old woman with a tuberculous granuloma. A transverse non-enhanced CT image shows a round SN (41 HU) located in the right lower lobe (**e**), which has rim enhancement on the CECT image (**f**). A 66-year-old woman with necrotizing granuloma. A transverse non-enhanced CT image shows a round SN (10 HU) located in the right upper lobe (**g**), which has mild and heterogeneous enhancement (▵CT value = 20 HU) on the CECT image (**h**). A 47-year-old woman with adenocarcinoma. A transverse non-enhanced CT image shows a round SN (26 HU) located in the right upper lobe (**i**), which has moderate and heterogeneous enhancement (▵CT value = 48 HU) on the CECT image (**j**). A 74-year-old woman with cryptococcal granuloma. A transverse non-enhanced CT image shows an oval SN (22 HU) located in the left upper lobe (**k**), which has severe and homogeneous enhancement (▵CT value = 86 HU) on the CECT image (**l**). CT, computed tomography; SN, solid nodule; CECT, contrast-enhanced computed tomography

Two thoracic radiologists (H.-b.X. and Z.-g.C.) evaluated the lesions on axial, multi-planar reconstruction (MPR), and maximum intensity projection (MIP) images. In case of disagreement, a consensus was reached after a joint discussion and/or consultation with a third senior radiologist. The CT features of SNs were evaluated in the following aspects: distribution (upper lobes, or other lobes), size (the mean of the longest diameter and the perpendicular diameter on axial CT images), shape (round, oval, or irregular), margin (smooth or coarse), lobulation, spiculation, air bronchogram, pleural indentation, mediastinal and hilar lymphadenopathy, CT value (non-enhanced, AP, and VP images), enhancement characteristics (no significant enhancement, rim enhancement, enhancement with well-defined necrosis, homogeneous enhancement, and heterogeneous enhancement), and degree of enhancement for lesions with homogeneous or heterogeneous enhancement (mild, moderate, and severe). Lobulation was defined as an abrupt protrusion in the contour of the lesion [12]. Spiculation was defined as linear strands extending from the nodule surface into the lung parenchyma without reaching the pleural surface [12]. Air bronchogram was defined as an air-filled bronchi seen as radiolucent in the lesion, branching bands within pulmonary densities [12]. The pleural indentation was defined as the indentation or drawing in of the pleura, the thin tissue covering the lungs and lining the interior chest wall [12]. Mediastinal and hilar lymphadenopathy was defined as those with a diameter of more than 10 mm in the short axis on chest CT scans [12]. The enhancement patterns of lesions were first assessed by integrating non-enhanced, AP and VP images. All Hounsfield unit measurements were performed at mediastinal window settings on transverse images to ensure that partial volume averaging was minimized [13]. For lesions with homogeneous enhancement, a circular or ovoid region of interest (ROI) was placed at the level of the largest transverse diameter of the lesion, and the ROI diameters were approximately 70% of the nodule’s short- and long-axis diameters. In heterogeneous lesions, the ROI encompassed only the most avidly enhancing area, avoiding significant vessels and low-enhancement areas. ROI consistency was ensured through the copy-paste function of the workstation, maintaining identical dimensions and spatial position across all phases. The enhancement degree of nodules was quantified using ▵CT values: (1) ▵CT_A_: difference of CT values measured on AP and non-enhanced images; (2) ▵CT_V_: difference of CT values measured on VP and non-enhanced images; and (3) ▵CT_P_: difference of the peak CT value measured on either AP or VP images and CT value on non-enhanced CT images. No significant enhancement was defined as a ▵CT value < 15 HU [13]. Rim enhancement was defined as a nodule exhibiting a central portion without enhancement surrounded by a significantly enhanced peripheral ring on CECT [14]. Enhancement with well-defined necrosis was characterized by a focal area within the nodule that showed no enhancement and had a clear boundary, while the remaining portion exhibited homogeneous or heterogeneous enhancement. For nodules with homogeneous and heterogeneous enhancement, we further divided them into mild (≥ 15 HU but < 30 HU), moderate (≥ 30 HU but < 60 HU), and severe (≥ 60 HU) enhancement according to the ▵CT value (Fig. 2).

### Statistical analysis

Clinical data and CT features were statistically analyzed for each patient. Continuous variables were expressed as mean ± standard deviation (SD) or median (interquartile range, IQR), while categorical variables were presented as numbers and percentages. The intraclass correlation coefficient (ICC) and Cohen’s kappa coefficient were employed to assess interobserver agreement for continuous and categorical variables, respectively [15]. The Mann–Whitney *U*-test was utilized to compare continuous variables in clinical and CT features between granulomas and PLCs, and the Pearson chi-square test was used to compare categorical variables between these two groups. Logistic regression analysis using clinical and CT features was conducted to develop predictive models for granulomas. Receiver operating characteristic (ROC) analysis was conducted to determine optimal threshold values of age and the non-enhanced CT value for diagnostic performance assessments, and to evaluate the diagnostic performance of regression models. The DeLong test was used to compare the AUCs (area under the curves) of different logistic regression models. Differences with *p*-values less than 0.05 were considered statistically significant.

## Results

### Patients’ clinical characteristics and pathological natures of lesions

Among 183 patients with granulomas, 17 (9.3%) had multiple lesions (13 with 2 nodules and 4 with 3 nodules), of whom 9 (52.9%) had lesions within the same lobe and 5 (29.4%) had lesions with similar features. In contrast, among 196 patients with PLCs, 8 (4.1%) had 2 nodules, of whom 6 (75.0%) had lesions distributed in the same lobe and 1 (12.5%) had lesions demonstrating similar features. Of the 204 granulomas, 135 (66.2%) were tuberculosis (TB), and 32 (15.7%) were fungal infections, while the remaining 37 (18.1%) cases were determined as inflammatory granulomas but without a determined etiology due to lack of further examination. In comparison, the 204 PLC cases included 163 (79.9%) adenocarcinomas, 30 (14.7%) squamous cell carcinomas, 6 (2.9%) large cell lung cancers, 3 (1.5%) small cell lung cancers, and 2 (1.0%) typical carcinoids.

Table 1 summarizes the patients’ clinical characteristics. Patients with granulomas were significantly younger than those with PLCs (*p* < 0.001). History of diabetes (23.0% vs 9.2%) was significantly more prevalent in patients with granulomas (*p* < 0.001), while history of COPD (32.7% vs 15.3%) was more common in those with PLCs (*p* < 0.001). Compared to patients with granulomas, cases with increased cytokeratin 19 (31.1% vs 21.0%) and CEA (17.5% vs 4.5%) were all more common in patients with PLCs (each *p* < 0.05). ROC analysis revealed the optimal cutoff value of patient age for distinguishing granulomas from PLCs was ≤ 63 years.

**Table 1:** Patients’ clinical characteristics

Parameters	Patients with granulomas	Patients with PLCs	*p*-value
	(*n* = 183)	(*n* = 196)	
Age (years)	55.01 ± 11.03	63.23 ± 9.92	< 0.001
Gender (male)	119 (65.0)	110 (56.1)	0.076
History of hypertension	40 (21.9)	59 (30.1)	0.068
History of diabetes	42 (23.0)	18 (9.2)	< 0.001
History of COPD	28 (15.3)	64 (32.7)	< 0.001
History of malignant tumor	13 (7.1)	20 (10.2)	0.285
Smoking history	71 (38.8)	80 (40.8)	0.688
Drinking history	52 (28.4)	45 (23.0)	0.224
Cases with clinical symptoms	67 (36.6)	64 (32.7)	0.418
Types of surgery			
Lobectomy	9 (4.9)	181 (92.3)	< 0.001
Segmentectomy	30 (16.4)	9 (4.6)	< 0.001
Wedge resection	144 (78.7)	6 (3.1)	< 0.001
Laboratory parameters^∗^			
Increased white blood cell count	4 (2.5)	6 (3.3)	0.758
Increased neutrophil count	7 (4.5)	9 (4.9)	0.851
Increased lymphocyte count	3 (1.9)	6 (3.3)	0.514
Increased monocyte count	14 (8.9)	23 (12.6)	0.289
Increased CA 19-9	2 (1.3)	5 (2.7)	0.458
Increased pro-GRP	15 (9.6)	11 (6.0)	0.215
Increased cytokeratin 19	33 (21.0)	57 (31.1)	0.038
Increased CEA	7 (4.5)	32 (17.5)	< 0.001

*PLCs* peripheral lung cancers, *COPD* chronic obstructive pulmonary disease, *CA 19-9* carbohydrate antigen 19-9, *pro-GRP* pro-gastrin-releasing peptide, *CEA* carcinoembryonic antigen

^∗^ Only the patients with this data were compared, including 157 patients with granulomas and 183 with PLCs, and they were not included in the multivariate analysis. Data are expressed as a number (percentage) or mean ± SD

### Interobserver agreement of CT features

Table S1 summarizes the interobserver agreement for the CT features of the lesions. For the continuous variables, agreement for the non-enhanced CT value and the ▵CT value was good (0.750–0.890), and that for nodule size was excellent (≥ 0.900). For the categorical indicators, agreement for shape and air bronchogram was substantial (0.610–0.800), and those for other features were almost perfect (0.810–1.000) [15].

### The non-enhanced CT features of lesions

Table 2 summarizes the non-enhanced CT features of lesions. Granulomas were more frequently demonstrated irregular shape (27.0% vs 13.2%) and smooth margin (55.4% vs 38.2%) (each *p* < 0.05), whereas PLCs more frequently showed lobulation (58.8% vs 34.3%), air bronchogram (53.9% vs 35.8%), and pleural indentation (57.4% vs 39.7%) (each *p* < 0.05). Additionally, granulomas exhibited lower density in non-enhanced CT than PLCs (median [IQR], 14.50 [7.00–24.00] HU vs 28.00 [20.00–35.00] HU, *p* < 0.001). ROC analysis revealed the optimal cutoff value of the non-enhanced CT value for distinguishing granulomas from PLCs was ≤ 21 HU.

**Table 2:** The non-enhanced CT features of lesions

Parameters	Granulomas	PLCs	*p*-value
	(*n* = 204)	(*n* = 204)	
Location (upper lobes)	105 (51.5)	109 (53.4)	0.692
Size (mm)^∗^	15.15 ± 5.58	15.22 ± 4.94	0.576
Shape (irregular)	55 (27.0)	27 (13.2)	0.001
Margin (smooth)	113 (55.4)	78 (38.2)	0.001
Lobulation	70 (34.3)	120 (58.8)	< 0.001
Spiculation	79 (38.7)	92 (45.1)	0.192
Air bronchogram	73 (35.8)	110 (53.9)	< 0.001
Pleural indentation	81 (39.7)	117 (57.4)	< 0.001
Lymph node enlargement	44 (21.6)	45 (22.1)	0.905
Non-enhanced CT value (HU)^†^	14.50 (7.00–24.00)	28.00 (20.00–35.00)	< 0.001

Data of categorical variables are expressed as a number (percentage)

*CT* computed tomography, *PLCs* peripheral lung cancers

^∗^ Data are expressed as mean ± SD

^†^ Data are expressed as median (IQR)

### The CECT features of lesions

Table 3 summarizes the patterns of enhancement on CECT images. Granulomas more frequently showed no significant enhancement (30.4% vs 0%), rim enhancement (15.2% vs 1.0%), and enhancement with well-defined necrosis (18.6% vs 1.0%) (each *p* < 0.001), while PLCs exhibited a higher rate of heterogeneous enhancement (85.8% vs 18.1%, *p* < 0.001). A total of 73 granulomas and 200 PLCs with homogeneous and heterogeneous enhancement were analyzed to evaluate the degree of enhancement. No significant differences in CT values were observed between the 73 granulomas and 200 PLCs exhibiting homogeneous or heterogeneous enhancement, either in AP images (54.00 [41.00–73.00] HU vs 55.00 [39.25–72.00] HU) or VP images (61.00 [40.00–83.00] HU vs 66.50 [53.00–76.75] HU) (each *p* > 0.05). Among the lesions with homogeneous and heterogeneous enhancement, based on ▵CT_P_, granulomas had significantly higher incidences of mild enhancement (30.1% vs 11.5%) and severe (47.9% vs 10.0%) enhancement (each *p* < 0.001), whereas PLCs more frequently showed moderate enhancement (78.5% vs 21.9%, *p* < 0.001); based on ▵CT_A_, granulomas demonstrated a higher incidence of severe enhancement (24.7% vs 4.0%, *p* < 0.001) and PLCs more frequently exhibited moderate enhancement (46.5% vs 24.7%, *p* = 0.001), while no significant difference was observed in mild enhancement between them (50.7% for granulomas vs 49.5% for PLCs, *p* = 0.862); based on ▵CT_V_, granulomas demonstrated a greater incidence of mild enhancement (37.0% vs 20.5%; *p* = 0.001) and severe enhancement (34.2% vs 8.0%, *p* < 0.001), while PLCs more frequently exhibited moderate enhancement (71.5% vs 28.8%, *p* < 0.001).

**Table 3:** Patterns of enhancement on CECT images

Patterns of enhancement	Granulomas	PLCs	*p*-value
	(*n* = 204)	(*n* = 204)	
No significant enhancement	62 (30.4)	0 (0)	< 0.001
Homogeneous enhancement	36 (17.6)	25 (12.3)	0.127
Heterogeneous enhancement	37 (18.1)	175 (85.8)	< 0.001
Enhancement with well-defined necrosis	38 (18.6)	2 (1.0)	< 0.001
Rim enhancement	31 (15.2)	2 (1.0)	< 0.001

Data are expressed as a number (percentage)

*CECT* contrast-enhanced computed tomography, *PLCs* peripheral lung cancers

### Logistic regression model

The regression models were constructed based on the clinical characteristics (patients’ age, history of diabetes, and history of COPD), non-enhanced CT features (lesion shape, margin, lobulation, air bronchogram, pleural indentation, and non-enhanced CT value), and CECT features (lesion enhancement pattern) with statistical differences. Model A was developed to predict granulomas using clinical and non-enhanced CT features. On the basis of model A, models B, C, and D additionally incorporated enhancement patterns based on enhancement characteristics of lesions and degree of enhancement, respectively determined by ▵CT_A_, ▵CT_V_, and ▵CT_P_. The enhancement patterns of lesions were divided into moderate enhancement and non-moderate enhancement (no significant enhancement, rim enhancement, enhancement with well-defined necrosis, mild enhancement, and severe enhancement).

**Fig. 3:**
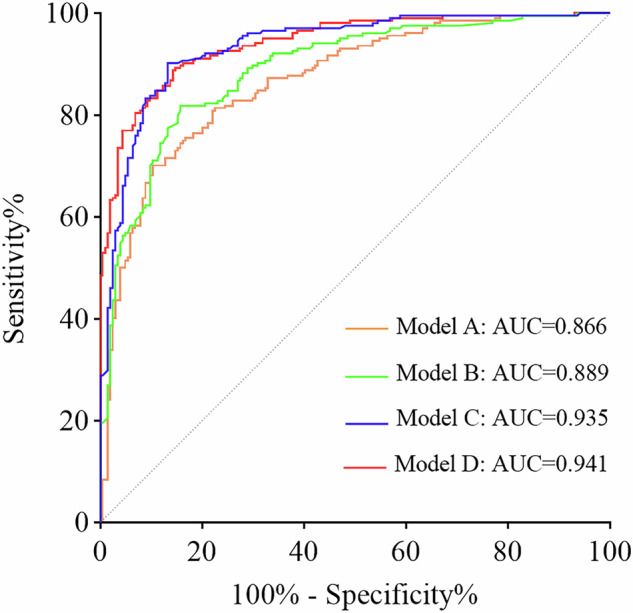
ROC curves of models A, B, C, and D. Model A was developed to predict granulomas using clinical and non-contrast CT features. On the basis of model A, models B, C, and D additionally incorporated enhancement patterns based on enhancement characteristics of lesions and degree of enhancement, respectively determined by ▵CT_A_, ▵CT_V_, and ▵CT_P_. Comparison of diagnostic performance: model A vs model B, model A vs model C, model A vs model D, model B vs model C, and model B vs model D (each *p* < 0.05); model C vs model D, *p* = 0.495

**Fig. 4:**
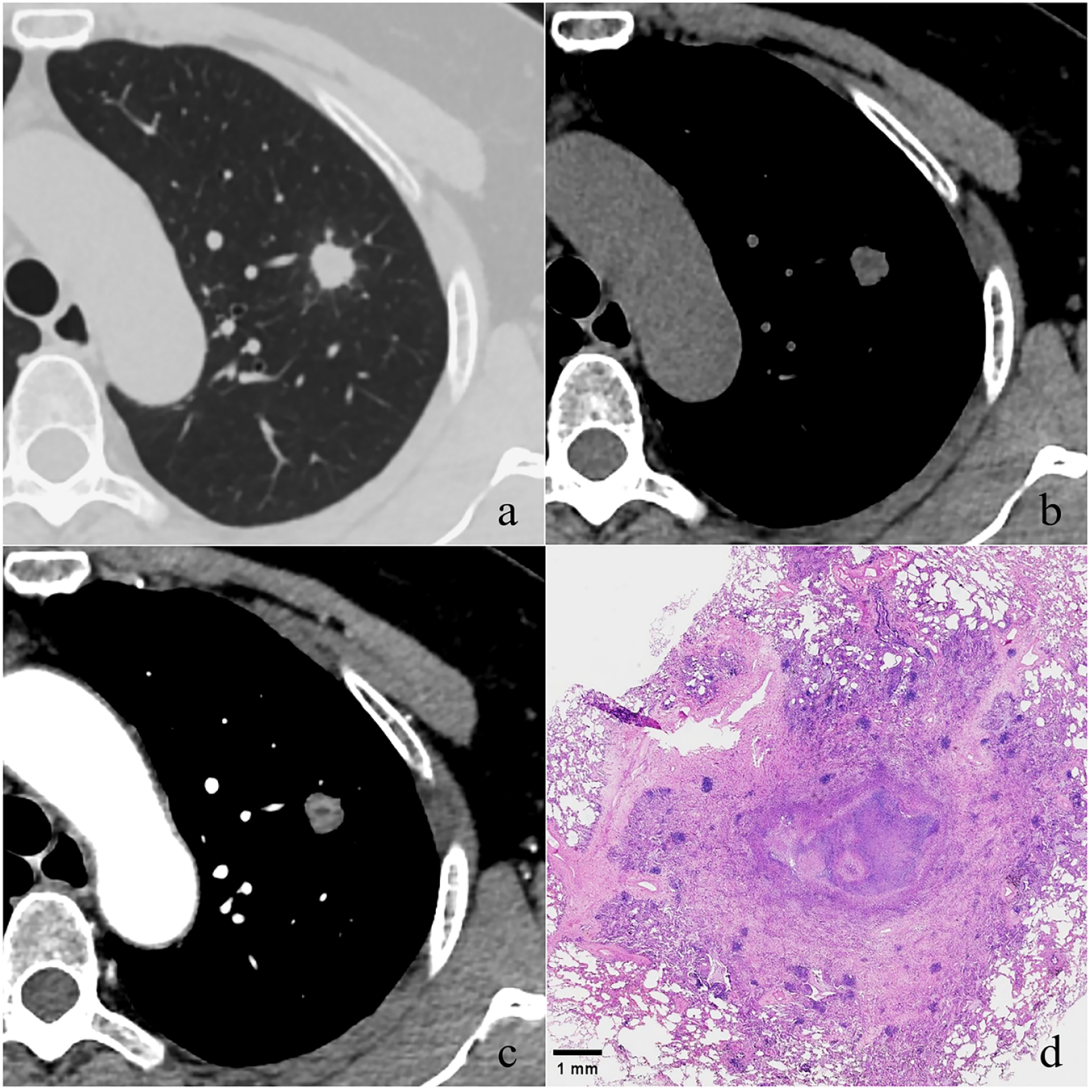
A 57-year-old woman with a 3-year history of diabetes and an 18-year history of hypertension. The transverse thin-section CT image shows a 10.4 mm round SN with spiculation located in the left upper lobe (**a**). This nodule exhibits a mean CT value of 13 HU on the non-enhanced image (**b**), and shows heterogeneous enhancement with well-defined necrosis in the central area on the CECT image (**c**). After the operation, it was confirmed as a tuberculous granuloma. Photomicrograph (H and E, ×6) shows there is significant central necrosis, other areas of the lesion exhibit extensive inflammatory cell infiltration and fibrosis (**d**). CT, computed tomography; SN, solid nodule; CECT, contrast-enhanced computed tomography

**Fig. 5:**
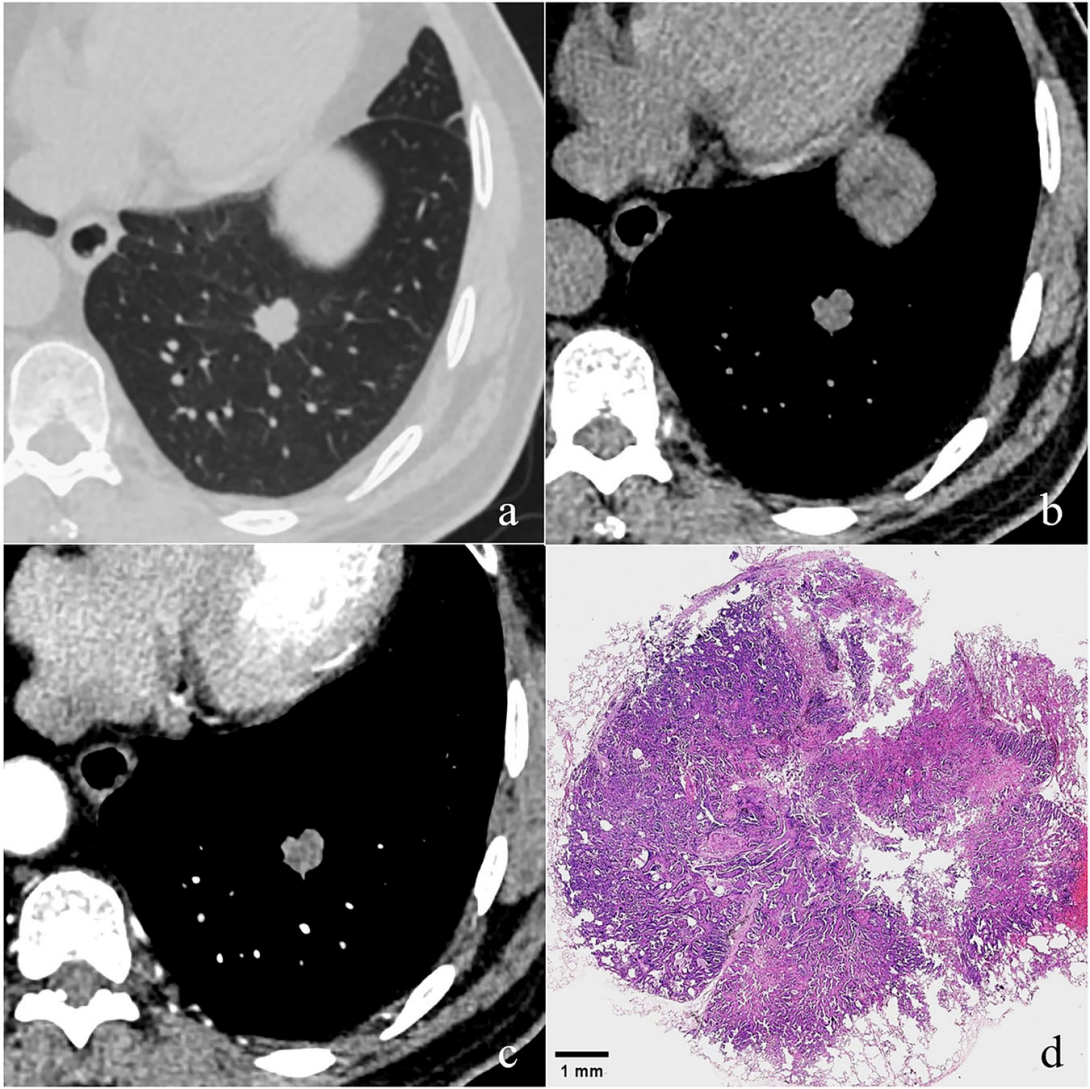
A 71-year-old man with a 12-year drinking history and a 22-year history of hypertension. Transverse thin-section CT shows a 15.1 mm round SN with lobulation located in the left lower lobe (**a**). This nodule exhibits a mean CT value of 26 HU on the non-enhanced image (**b**), and shows moderate and heterogeneous enhancement (▵CT value = 39 HU) on the CECT image (**c**). After the operation, it was confirmed as invasive adenocarcinoma. Photomicrograph (H and E, ×6) shows tumor cells within the nodule exhibiting both lepidic and acinar growth patterns, along with a thickened alveolar septum and localized fibrosis (**d**). CT, computed tomography; SN, solid nodule; CECT, contrast-enhanced computed tomography

Compared with model A (AUC: 0.866, 95% confidence interval [CI]: 0.831–0.901; sensitivity: 76.5%, specificity: 81.4%), the diagnostic performances of model B (AUC: 0.889, 95% CI: 0.858–0.921; sensitivity: 81.4%, specificity: 81.9%), model C (AUC: 0.935, 95% CI: 0.911–0.959; sensitivity: 87.3%, specificity: 84.8%), and model D (AUC: 0.941, 95% CI: 0.919–0.962; sensitivity: 88.7%, specificity: 83.8%) significantly increased (each *p* < 0.05). Pairwise comparisons among models B, C, and D revealed that only model C demonstrated diagnostic performance comparable to model D (*p* = 0.495) (Fig. 3). Moreover, model D revealed that age ≤ 63 years (odds ratio [OR], 5.237; 95% CI: 2.609–10.509), history of diabetes (OR, 9.097; 95% CI: 3.056–27.077), irregular shape (OR, 3.603; 95% CI: 1.594–8.142), non-enhanced CT value ≤ 21 HU (OR, 7.576; 95% CI: 3.720–15.431), and non-moderate enhancement (OR, 50.065; 95% CI: 20.293–123.517) were independent indicators for predicting granulomas (Table 4 and Figs. 4 and 5).

**Table 4:** Logistic regression analysis for predicting granulomas from PLCs

Parameters	OR (95% CI)	*p*-value
Age (years)		< 0.001
> 63	1	
≤ 63	5.237 (2.609, 10.509)	
History of diabetes		< 0.001
No	1	
Yes	9.097 (3.056, 27.077)	
History of COPD		0.019
No	1	
Yes	0.397 (0.183, 0.860)	
Shape (irregular)		0.002
No	1	
Yes	3.603 (1.594, 8.142)	
Lobulation		0.044
No	1	
Yes	0.520 (0.275, 0.982)	
Non-enhanced CT value (HU)		< 0.001
> 21	1	
≤ 21	7.576 (3.720, 15.431)	
Non-moderate enhancement^∗^		< 0.001
No	1	
Yes	50.065 (20.293, 123.517)	

*PLCs* peripheral lung cancers, *OR* odds ratio, *CI* confidence interval, *COPD* chronic obstructive pulmonary disease

^∗^ Non-moderate enhancement is defined as no enhancement, annular enhancement, enhancement with focal necrosis, or homogeneous or inhomogeneous enhancement exhibiting a mild or severe degree

## Discussion

This study comprehensively compared the clinical and CT features of atypical granulomas and PLCs, revealing significant differences between these two entities. It was found that some traditional clinical and morphological features of PLCs were not effective for differentiation. In contrast, indicators such as patient age, history of diabetes, history of COPD, lesion shape, lobulation, non-enhanced CT value, and enhancement patterns served as important clues for accurate diagnosis. Specifically, SNs that exhibited lower non-enhanced CT value (≤ 21 HU), irregular shape, and non-moderate enhancement in younger patients (≤ 63 years) with diabetes should first be suspected as granulomas. Given that enhancement features could provide critical information, CECT should be preferred for differentiating them.

Recent studies on diagnostic models for differentiating granulomas from lung cancer have primarily focused on machine learning approaches. Lin et al [16] developed a deep learning model based on radiomic features, achieving an AUC of 0.871, which improved to 0.907 after integrating clinical and imaging features. Beig et al [17] reported lower AUCs of 0.80 and 0.76 based on radiomic and deep learning models, respectively, for distinguishing non-calcified granulomas from lung adenocarcinomas. In contrast, this study established a diagnostic model integrating clinical and CT features to differentiate atypical granulomas from PLCs, with the highest AUC of 0.941, outperforming prior machine learning models. This improvement may be attributed to the fact that previous studies predominantly analyzed single-phase CT images (either non-contrast or contrast-enhanced), thereby lacking critical enhancement characteristics. In contrast, the present study integrates non-enhanced and contrast-enhanced CT features, enabling more precise characterization of granulomas that exhibit significant pathological heterogeneity across different disease stages.

Studies indicated that clinical risk factors for lung cancer include older age, histories of smoking and COPD, and elevated tumor markers [18, 19]. In this study, patients with PLCs were older and more frequently exhibited COPD, along with higher levels of cytokeratin 19 and CEA, consistent with previous results [20, 21]. However, a history of smoking was not a predictor of PLCs, possibly because it was also a risk factor for granulomas [8, 22]. Additionally, this study identified diabetes as a risk factor for granulomas, which may be related to the fact that granulomas, often caused by TB and fungal infections, are more likely to develop in patients with immunocompromised conditions due to diabetes [11, 23]. Therefore, when differentiating granulomas and PLCs, it is essential to consider the patients’ clinical data, particularly the presence of potential immunosuppressive conditions.

Regular SNs with lobulation were highly indicative of lung cancer, especially those with coarse margin, air bronchogram, spiculation, and pleural indentation [1, 12, 24]. In this study, irregular SNs without lobulation were found to be relatively common in granulomas, which is similar to the previous study focused on comparing benign and malignant pulmonary nodules [24]. However, other CT morphological features were not reliable predictors for either granulomas or PLCs. Furthermore, this study found that it was not effective for mediastinal and hilar lymphadenopathy in distinguishing PLCs from granulomas, inconsistent with previous studies on benign and malignant nodules [25]. This may be because both inflammation caused by granuloma and lung cancer metastasis can lead to lymphadenopathy [8, 26]. Consequently, it is challenging to differentiate them based solely on the CT morphological features. Thiessen et al [7] obtained similar results, they found that CT morphological features were insufficient to differentiate granulomas with necrosis from malignant tumors, requiring core needle biopsy for definitive diagnosis. However, the risk of false-negative results associated with needle biopsy necessitates caution [27]. Therefore, integrating additional valuable CT features with biopsy validation is essential, particularly for those stable SNs during follow-up.

On non-enhanced CT images, it was found that granulomas more frequently exhibited lower density (≤ 21 HU), which may be closely associated with their tendency for necrosis [8, 28]. Thus, the non-enhanced CT value of lesions could be considered as a potentially valuable predictive indicator in distinguishing between granulomas and PLCs. This finding has not been highlighted in the differential diagnosis of benign and malignant nodules in previous studies. It suggests that for clinically indeterminate SNs, measuring the CT value on non-enhanced CT images is essential. A lower non-enhanced CT value indicates a greater likelihood that the nodule is a granuloma.

Various enhancement patterns observed on CECT images are useful in differentiating pulmonary SNs. This study found that granulomas displayed five distinct patterns of enhancement, which may be closely related to their pathological changes at different stages, offering crucial clues for better differentiation. Granulomas in the active phase typically exhibit a rich blood supply, which diminishes as they mature due to vascular regression and necrosis [13, 29]. Thus, they may show severe enhancement, mild enhancement, and no significant enhancement, consistent with previous findings [13, 30]. Additionally, due to frequent necrosis, they may initially present significant enhancement accompanied by a well-defined area without enhancement due to focal necrosis, and subsequently exhibit rim enhancement due to a larger area of necrosis surrounded by fibrotic tissue [14].

In contrast to granulomas, the PLCs mainly displayed moderate (30–59 HU) and heterogeneous enhancement. Similar results were acquired by Swensen et al [13] and Ye et al [30], who found that the mean ▵CT values of lung cancers were 38.1 HU and 38.7 HU, respectively. Another study by Li et al [31] also revealed that lung cancers frequently exhibit heterogeneous CT perfusion. Pathologically, the newly formed networks of blood vessels in lung cancers increase the tumor blood supply, which is generally lower than that caused by the vasodilatation in active granulomas [32]. Furthermore, ischemic necrosis and fibrosis mixed within the tumor usually lead to uneven blood supply [31, 33]. These characteristics resulted in the relatively uniform enhancement patterns of PLCs.

This study has several limitations that should be considered. Firstly, as a retrospective single-center study, our findings are constrained by the limited sample size. Future multicenter studies with larger cohorts are required to validate these results, and systematic comparison of different machine learning models is also warranted to enhance the robustness of diagnostic predictions. Secondly, the current results on granulomas were primarily based on TB nodules. Therefore, the applicability of our conclusions may be geographically limited, as TB may not be the common cause of granulomas in some regions. Thirdly, the specific etiology of some granulomas remains unclear, primarily due to the retrospective nature of the study. Finally, the analysis and comparison were limited to atypical granulomas and PLCs, so the findings are specifically focused on distinguishing between these two types of lesions. Fourthly, our diagnostic model in this study was developed based on dynamic three-phase images. However, since this approach is not routinely performed in some institutions, the application of the present findings may be limited. After comparison, the diagnostic model based on the VP performed comparably to the dynamic three phases, and the enhancement characteristics of lesions could also be well observed on VP images. Therefore, the VP can be solely used instead of both the arterial and VPs. Finally, the variations in tube voltage settings (110–130 kV) across CT scanners may influence measurements of CT values, while there was no significant difference in the use of kV values between granulomas and PLCs.

## Conclusion

Atypical granulomas closely mimic PLCs in morphological features, posing a great challenge in differentiation. CECT scans could provide important information about the lesions, which is crucial for their differential diagnosis. In younger (≤ 63 years) patients with diabetes, an irregular SN exhibiting lower density (≤ 21 HU) in non-enhanced CT and a non-moderate enhancement pattern should first be considered as granulomas. Understanding the clinical and CT characteristics of granulomas and PLCs is beneficial for accurately identifying them and reducing unnecessary surgical resections.

## Supplementary information

Supplementary information

## Data Availability

The datasets generated during the current study are not publicly available due to our institutional regulations, but are available from the corresponding author on reasonable request.

## Abbreviations

AP: Arterial phase
AUCs: Area under the curves
CA 19-9: Carbohydrate antigen 19-9
CEA: Carcinoembryonic antigen
CECT: Contrast-enhanced computed tomography
CI: Confidence interval
COPD: Chronic obstructive pulmonary disease
CT: Computed tomography
IQR: Interquartile range
OR: Odds ratio
PLCs: Peripheral lung cancers
pro-GRP: Pro-gastrin-releasing peptide
ROC: Receiver operating characteristic
ROI: Region of interest
SD: Standard deviation
SNs: Solid nodules
SSNs: Sub-solid nodules
TB: Tuberculosis
VP: Venous phase
