# Supplementary material for: Exploring the key clinical and CT characteristics of granulomas mimicking peripheral lung cancers: a case-control study
Source: Insights Imaging. 2025 Jul 19;16:157. doi: 10.1186/s13244-025-02043-0 (PMC12276186; doi:10.1186/s13244-025-02043-0)
Supplement: Supplementary file 1 — Supplementary information [file 13244_2025_2043_MOESM1_ESM.docx]

**Table S1** Interobserver agreement of CT features

| **Parameters** | **Metric**^*^ | **95% CI** |
| --- | --- | --- |
| Size | 0.938 | 0.888-0.961 |
| Shape | 0.745 | 0.667-0.823 |
| Margin | 0.819 | 0.764-0.874 |
| Lobulation | 0.887 | 0.842-0.932 |
| Spiculation | 0.888 | 0.843-0.933 |
| Air bronchogram | 0.784 | 0.723-0.845 |
| Pleural indentation | 0.823 | 0.768-0.878 |
| Lymph node enlargement | 0.878 | 0.821-0.935 |
| Non-enhanced CT value (HU) | 0.824 | 0.777-0.860 |
| △CT value (HU) | 0.805 | 0.702-0.866 |
| Patterns of enhancement | 0.864 | 0.823-0.905 |

Table S1: * Metric represents ICC for continuous variables and kappa coefficient for categorical variables. Interobserver agreement, as indicated by ICC, was classified as poor (< 0.500), moderate (0.500−0.740), good (0.750−0.890), or excellent (≥ 0.900) [15]. The agreement based on kappa coefficients was categorized as poor (< 0.000), slight (0.000−0.200), fair (0.210−0.400), moderate (0.410−0.600), substantial (0.610−0.800), or almost perfect (0.810−1.000) [15]. CT, computed tomography; CI, confidence interval; ICC, intraclass correlation coefficient.
